# Exploring Patients’ Feeling of Being Coerced During Psychiatric Hospital Admission: A Qualitative Study

**DOI:** 10.1007/s11126-023-10039-6

**Published:** 2023-07-15

**Authors:** Benedetta Silva, Mizué Bachelard, Charles Bonsack, Philippe Golay, Stéphane Morandi

**Affiliations:** 1 Community Psychiatry Service, Department of Psychiatry, Lausanne University Hospital and University of Lausanne, Lausanne, Switzerland; 2 Cantonal Medical Office, General Directorate for Health, Canton of Vaud Department of Health and Social Action, Lausanne, Switzerland; 3 General Psychiatry Service, Department of Psychiatry, Lausanne University Hospital and University of Lausanne, Lausanne, Switzerland; 4 Institute of Psychology, Faculty of Social and Political Sciences, University of Lausanne, Lausanne, Switzerland

**Keywords:** Coercion, Experience, Psychiatric hospitalisation, Qualitative study, Thematic analysis

## Abstract

Various coercive measures can be used to legally compel a person suffering from psychiatric disorder to undergo treatment. However, evidence suggests that patients’ feeling of being coerced is not determined solely by their being submitted to formal coercion. This study aimed to explore voluntary and involuntary patients’ experience of coercion during psychiatric hospitalisation and to identify which factors, from their perspective, most affected it. We chose a qualitative design inspired by a hermeneutic-phenomenological approach. Participants were purposively selected from six psychiatric hospitals in Switzerland. Maximum variation sampling was used to ensure the inclusion of patients with different levels of perceived coercion and different admission statuses. In-depth, semi-structured interviews were co-conducted by a research psychologist and a service-user researcher. The transcribed data underwent thematic analysis. All twelve interviewed patients described the hospitalisation as an experience of loss of control over their life due to either external or internal pressures. During the process, perceptions of these pressures varied and sometimes overlapped, leading some patients to describe their admission as a form of simultaneous protection and violation. The balance between these two contradictory feelings was affected by a variety of contextual and relational factors, as well as by the meaningfulness of the experience and the patient’s subsequent satisfaction with it. Increasing policy-makers’ and clinicians’ awareness about the main factors influencing patients’ experience of loss of control is of paramount importance in order to develop skills and strategies able to address them, reinforcing patients’ empowerment, reducing their feeling of coercion and improving their well-being.

## Introduction

Coercion is defined as “the practice of persuading someone to do something by using force or threats” [1]. In psychiatry, various coercive procedures can be used to legally compel a person to undergo treatment. These practices—also referred to as formal coercive measures—include involuntary admission, seclusion, restraint and forced medication. In recent decades, several studies have examined formal coercion with the aim of reducing its use and negative impacts on the persons concerned. However, it has become increasingly clear that formal coercion is just the tip of the iceberg [2] and that “the boundary between coercive measures and patients’ voluntary acceptance of treatment is blurred” [3]. Evidence suggests that voluntary patients may feel just as coerced into treatment as involuntary patients, whereas the latter sometimes perceive no coercion despite the formal coercive measures they are subjected to. A literature review and meta-regression published in 2012 found that around 25% of legally detained patients did not feel coerced into psychiatric care, whereas the same percentage of voluntary inpatients did [4]. In 2017, the “Service Users’ Perspective of their Admission” study, conducted in Ireland, reported a similar result: 22% of voluntarily admitted patients felt the same levels of perceived coercion as those admitted involuntarily [5]. A recent study in Switzerland revealed that 6.6% of voluntary and 30.4% of involuntary patients reported an erroneous admission status and that the experienced levels of coercion were more strongly predicted by their perceived than their actual legal status [6].

Different forms of informal coercion, such as persuasion, interpersonal leverage, inducements or threats [7], can be used instead of or in addition to formal coercive measures, affecting the patient’s subjective experience. However, it is often unclear whether and when such interventions are deemed coercive or not [3, 8]. Wertheimer (1993) defined a “threat” as a conditional proposal that, if refused, would leave the person worse off in comparison to a “moral baseline”, e.g. depriving them of liberty. He contrasted this to a non-coercive “offer” whose refusal would not leave the person worse off [9]. For Sjostrom (2006), the “coercion context” was decisive in determining the coercive experience of both voluntary and involuntary patients. In such context, patients may perceive professionals’ predictions, opinions, advice, offers and expectations as threats [8, 10]. Fennell (2010) stated that “Compliance in the shadow of compulsion is an important feature of the psychiatric system” [11]. This “coercive shadow”—the fear that non-compliance will lead to the use of compulsion—is what pushes some patients to “voluntarily” agree to treatment [12]. Finally, several authors have recognised the critical role that the interpersonal process of *procedural justice* has on perceived coercion. Their studies confirmed that how voluntary and involuntary patients felt staff had treated them during the hospital admission process strongly affected their experience, in some cases even more than formal coercion itself [13–16].

A purely dichotomous model of coercion, opposing coercion with non-coercion, would therefore be misleading and result in an oversimplification of the phenomenon [3]. Researchers should instead use a broader definition of coercion to investigate a fuller range of experiences and focus more on its relational and contextual aspects [2, 17]. Qualitative approaches have been identified as the most useful methods for obtaining a deeper understanding of coercion as defined and experienced by patients [17, 18]. Several qualitative studies of patients’ feeling of being coerced during hospital admission have been published. However, a recent literature review on this topic highlighted that very few of them considered voluntary patients’ experience of coercion [19]. Previously, Seed et al. (2016) reviewed findings on experiences of involuntary admission and found that they mirrored those of inpatients’ experiences in general; they considered it important that future research consider both voluntary and involuntary patients [20]. Service users also emphasised that not limiting studies on coercion to involuntary patients was the only way to ensure that “all the single occurrences where people are denied a choice about basic things in lives” were taken into account [17].

This qualitative study aimed to explore voluntary and involuntary patients’ experience of coercion during psychiatric hospital stay and to identify which factors, from the participants’ perspective, most affected it. To the best of our knowledge, this was the first study to explore the experience of coercion among both voluntary and involuntary patients in Switzerland. The Swiss Confederation has one of the most highly developed psychiatric health systems in the world [21]. According to World Health Organization data, in 2014, Switzerland had 91 psychiatric beds per 100,000 inhabitants, well above the European average of 73 [22]. In 2021, 9.1 psychiatric hospitalisations per 1,000 inhabitants were reported in Switzerland, with an average length of stay of 34.2 days [23, 24]. Moreover, the Swiss Health Observatory reported that 1.9 involuntary admissions per 1,000 inhabitants were registered the same year [25], one of the highest rates in Europe [26]. Despite a proclaimed shift from hospital-based to more community-based mental healthcare, inpatient care remains at the core of Switzerland’s mental health system [27]. In this context, exploring patients’ experience of hospitalisation and their feeling of being coerced while inpatient seems of utmost relevance. A deeper understanding of how coercion, in all its forms, was experienced by patients and what, in their opinion, could have changed their experience is essential to developing interventions that could reduce this feeling and its well-known negative effects [28–32]. Furthermore, increased awareness among professionals of how patients perceive the most subtle forms of coercion could prompt them to consider their use more closely, thereby improving the quality of care and patient satisfaction.

## Methods

### Methodological Approach

We chose a qualitative design inspired by an interpretative hermeneutic-phenomenological approach in order to gain a deeper understanding of the nature and meaning of patients’ experience of coercion [33]. Drawing on the writings of Husserl, phenomenology seeks to capture a phenomenon’s meaning “through investigating and analysing lived examples of the phenomenon within the context of the participants’ lives” [34]. In the hermeneutic tradition, built upon the works of Heidegger and Gadamer, the meaning of the experience arise from the meeting between the researchers’ experiences and prior understandings and the subject’s experience of the phenomenon [35]. Thus, hermeneutic-phenomenology allow to achieve a balance between the phenomenological close description of the participants’ experience and the hermeneutic, self-reflexive investigation of the phenomenon [35].

### Recruitment and Participants

Participants were purposively selected from a larger cohort of 230 patients previously recruited for the quantitative arm of a prospective study on coercion and shared decision-making in six psychiatric hospitals in Switzerland’s French-speaking regions. Recruitment took place between April 2020 and July 2022. The inclusion criteria required the study’s patients to be aged between 18 and 65 years old, to have been hospitalised for more than seven days but less than 15, and to be able to provide their formal consent to participate. Patients affected by organic mental disorders or intellectual disabilities or insufficiently proficient in French were excluded. To gather a sample with comparable clinical histories, only patients with no more than five previous psychiatric hospital admissions were included in the study’s qualitative arm. On the contrary, maximum variation was sought for levels of perceived coercion at admission, as assessed using the validated French version of the MacArthur Admission Experience Survey (AES) Short Form [36], and for admission status (voluntarily and involuntarily admitted patients). Participants were recruited in three waves so as to enable a periodic adjustment of the sample’s characteristics and experiences based on the first emerging findings. Recruitment continued until saturation was reached.

At the end of their quantitative assessment, a research psychologist informed the patients screened for an interview about the study’s qualitative arm. Those who agreed to participate were asked to provide their contact details (telephone number, email, or postal address). A member of the research team contacted them again after their discharge, but not earlier than one month after their quantitative assessment, to verify whether they were still interested in participating in the study and, if so, schedule a meeting for the interview.

Twelve patients were interviewed for this study: three males and nine females aged between 19 and 61 years old. Five had been hospitalised previously, while for seven of them it was their first hospital admission. Three participants were diagnosed on admission as affected by personality disorders, two by schizophrenia, two by depressive mood disorders, two by anxiety disorders, one by bipolar disorder and one by alcohol-related mental disorder. Reasons for hospital admission as perceived and self-reported by participants during the interviews were: suicide attempt (two participants); suicidal ideation and self-harm risk (four participants); psychotic decompensation (two participant); psychic decompensation due to an organic problem (one participant) and anxiety crisis (one participant). Reasons for admission were unclear for two participants. Seven participants were admitted voluntarily and four involuntarily. One participant was placed under involuntary detention one day after their voluntary admission. One patient underwent a forced medication treatment during their hospitalisation, and another was both forcibly medicated and secluded.

### Data Collection

Data were gathered using in-depth, semi-structured interviews co-conducted by a research psychologist (B.S.) and a service-user researcher (M.B.). The service-user researcher’s presence during the interviews was meant to foster participants’ openness and manage the power imbalance between researchers and participants [37]. The interviewers used a flexible topic guide with open-ended questions focusing on the participants’ experience of hospitalisation, the coercion perceived throughout that process and the factors that most affected this experience. The initial topic guide was developed based on a literature review and the research team’s expertise. The guide was tested using three pilot patient interviews to ensure that the questions included were understandable and of interest from the patients’ points of view, and that the data produced were rich and of sufficient quality. After six interviews, the guide was adapted once more accordingly to the first results of the data analysis. All the interviews were audio-recorded, transcribed verbatim by a member of the research team and anonymised during that transcription. Participants were allowed to choose the location for their interview. Eight decided to be interviewed at the Community Psychiatry Service’s premises, while four preferred their home. Interviews were held between two and eight months after their hospital admission and lasted between 46 and 134 min. All interviews were conducted in French. The verbatim statements presented in this article were translated into English by a professional translator in order to ensure their authenticity and best capture their meaning.

### Data Analysis

Thematic analysis from Braun and Clarke (2006) was performed [38]. After familiarisation with the data through repeated readings, initial codes were generated. Patterns in codes were sought, and themes and sub-themes were created. Visual representations were used during this phase to help the sorting process. Themes were subsequently revised, and their internal homogeneity and external heterogeneity were assessed [39]. Extracts of each theme were reread as well as the entire dataset in order to verify theme coherence and to code potentially missed data. Finally, themes and sub-themes were defined and named based on their core meanings.

The analysis process was inductive, allowing the researchers to maintain an open mind about unexpected meanings, and cyclical [40]. Coding strategies were cross-checked by independent researchers. Two researchers (B.S. and M.B.) independently coded the first interview and compared their results. Any disagreements were discussed until agreement on the meaning and the application of each initial code was reached. Next, the two researchers independently coded another set of two interviews and an inter-rater reliability score was calculated (Cohen’s Kappa). All cases with a Cohen’s Kappa below 0.6 were discussed until an agreement was reached. The codebook was then independently reapplied to another set of three interviews. The cross-checking process ended when each code reached a mean Cohen’s Kappa above 0.6. One researcher (B.S.) coded the remaining interviews under the continuous supervision of another (M.B.). The coding framework was discussed and refined in research meetings with the whole team. NVivo (release 1.5) software was used for data analysis and storage.

### Ethical Considerations

The Human Research Ethics Committee of the Canton of Vaud approved the study (protocol #2016–00768). All potential interviewees were provided with a participant information sheet and given at least 24 hours to agree to take part in the study. A written consent form was signed before commencing each interview. Researchers were not involved in the participants’ care. Patients were informed that the persons responsible for the study had no links with the hospital staff and that their refusal to participate would in no way affect their care. They were also made aware that they were free to terminate the interview at any time or refuse to answer any question. Audio-recorded interviews were transcribed and any personal details that might help identify a participant were omitted. Each participant was attributed a pseudonym. After transcription, the audio files were destroyed. The study was carried out in accordance with the recommendations of the Human Research Ethics Committee of the Canton of Vaud and the Declaration of Helsinki.

### Researchers’ Reflexivity

Being aware of their role in the co-construction of meanings and their possible influence on data collection and analysis, the researchers adopted a reflexive posture throughout the process in an attempt to improve the study’s transparency and trustworthiness [37]. The first author (B.S.) is a researcher with a background in psychology and psychotherapy and a long experience in studies on coercion. The second author (M.B.) is a service-user researcher with a background in educational sciences and personal experience with mental health issues, hospital admissions and coercion. The researchers’ personal and professional experiences and research interests could have affected the analysis. To address this issue, the analysis was repeatedly discussed with the other research team members, including a professor of psychiatry (C.B.), a senior psychiatrist (S.M.) and a senior research psychologist (P.G.), and in interdisciplinary meetings including clinicians, researchers from other institutions, peer-practitioners and patients.

## Results

**Fig. 1:**
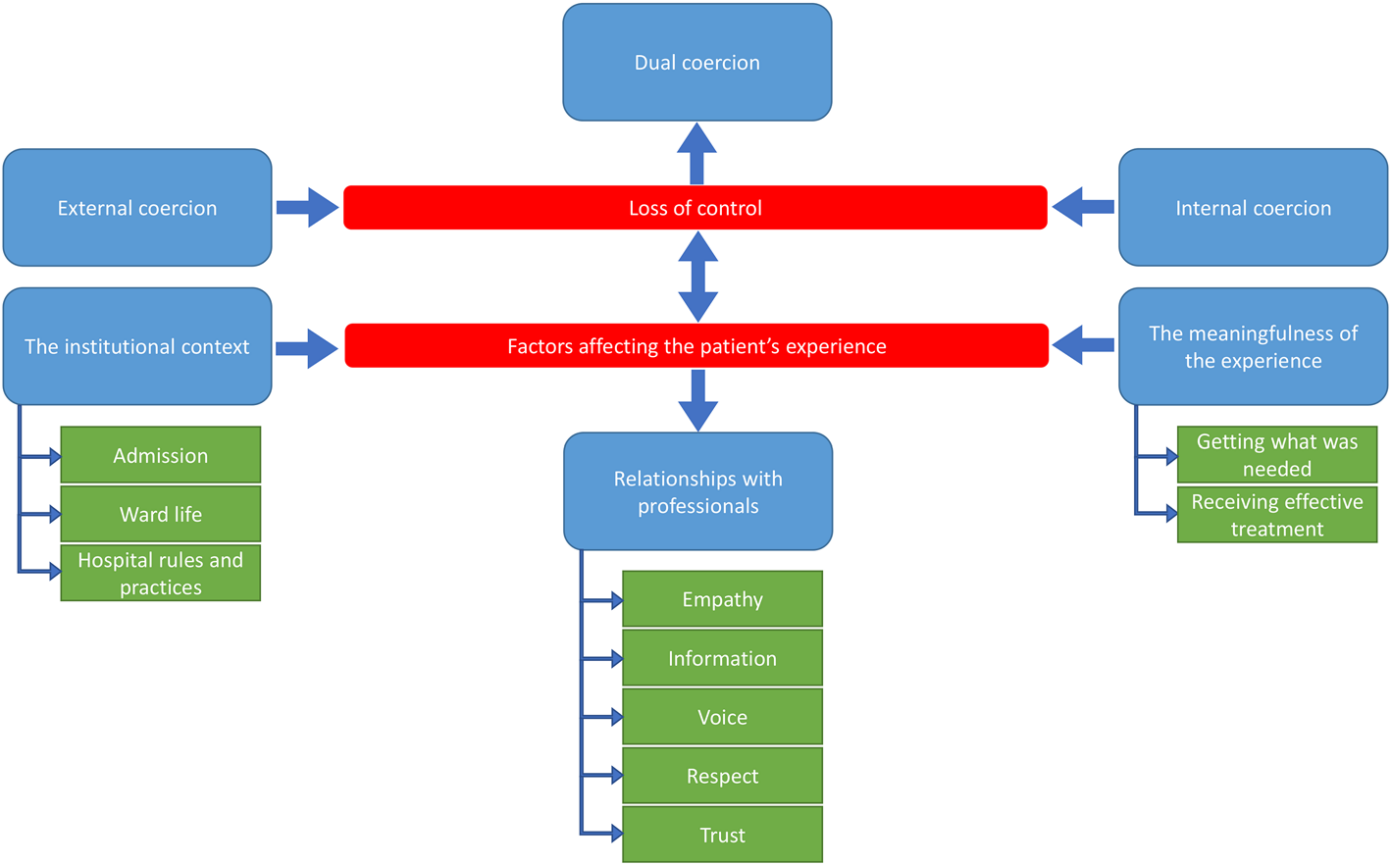
Patients’ feeling of being coerced during psychiatric hospital admission: thematic map of overarching themes, themes and sub-themes

Interview content was organised into two overarching themes. Overarching theme 1 was “*Loss of control”,* capturing the essence of the hospital experience as reported by patients. Overarching theme 2 was “*Factors affecting the experience”,* outlining the most important elements that, from patients’ viewpoints, influenced this feeling of loss of control throughout the entire hospitalisation process. Each overarching theme included three interconnected themes and several sub-themes. The overarching themes, themes and sub-themes are summarised in Fig. 1.

### Loss of Control

Patients described their hospitalisation as an experience of loss of control over their lives and choices. Some described this feeling as being mainly due to the intervention of an external authority that overpowered them and took decisions in their stead (*external coercion)*. For others, the main source of disempowerment was their mental condition, which in times of mental crisis, prevented them from maintaining any control over their lives (*internal coercion*). These two forms of pressure were not mutually exclusive. During the hospitalisation process, patients’ perceptions could vary and sometimes overlap, leading to a feeling of *dual coercion*.

#### External Coercion

During their hospitalisation process, some patients, whether voluntarily or involuntarily admitted, felt overpowered by an external authority that deprived them of their liberty and left them with no choices or alternatives:

*“…from that moment on* [hospital admission], *you fall under the authority of somebody else, and I get the feeling of not having a choice in the matter anymore!”* (Laura, IH)

That external authority was mainly represented by mental health care professionals. However, in specific situations, stakeholders such as family members, the police or other local authorities were also identified as key actors in depriving patients of their right to self-determination. That power could be exerted formally or informally. Persuasion, inducements and even threats were often mentioned as means of convincing them to voluntarily accept their hospital admission:

*“…administratively, it was voluntary; in reality, in the emergency room, the psychiatrist, or rather the junior doctor, told me that…well, she asked me if I was okay with going in, saying that it would be nicer for everyone if she didn’t have to section me, but that if I didn’t agree, she’d do it! So, it wasn’t really voluntary. It was a bit ‘Either you’re okay going in or I’ll force you to go!’”* (Alice, VH)

The use of *external coercion* was not limited to the admission phase. Similar pressures were reported throughout the hospitalisation process: to push patients into taking their medication, accepting the hospital’s restrictions and sometimes even adhering to post-discharge treatment plans:

*“…they came to see me to offer me a Temesta* [lorazepam], *and when I said that I didn’t want it, they told me, ‘It’s either an injection or you swallow a Temesta!’ So, you know, I didn’t even try to understand!”* (Laura, IH)

Although most of these patients acknowledged that their mental condition before their admission was fairly critical and that they needed help, they regarded the authority’s decision to hospitalise them as disproportionate, which generated strong feelings of anger, injustice, helplessness and fear:

*“…yeah, but then I wasn’t being auto or hetero-aggressive, I am not, so I said it; I really said it, and that’s also why I still haven’t understood the choice* [they made] *because there weren’t enough reasons for me to be sectioned!”* (François, IH)

*“It’s just that I felt as if I was being punished for nothing, you know? It’s just a feeling of helplessness!”* (Maude, VH)

Some patients’ initial reaction was to try to oppose the authorities. Patients resorted to verbal and even physical resistance in the attempts to make their voices heard, show their unhappiness and regain some control over the situation:

*“Well, I was very annoyed about being hospitalised, and so to protest that—because I was endlessly repeating that it wasn’t right—once I was in the room, I flipped over the table. I’d made a lot of noise, on purpose, really, to make them understand that this wasn’t what I wanted at all!”* (François, IH)

*“When I feel helpless, and I feel misunderstood… I start to raise my voice a little bit…because it isn’t easy to stay calm when you feel misunderstood!”* (Maude, VH)

However, they soon realised that expressing their opposition only led to even more restrictions. The only way to get out of this situation as soon as possible, be discharged and regain control over their lives was to surrender, conform to the system and behave as expected:

*“I think that I said the right things, having changed unit and getting a new senior doctor. I think I said the right things there. I behaved less… (…) As soon as I arrived in the unit upstairs, I did it straight away. (…) I quickly changed how I behaved, how I acted. Yeah, you adapt!”* (François, IH)

*“I wanted to disagree with it all, but then I told myself it wasn’t worth it! (…) Sometimes I just wanted to show that I was doing okay, too, so that I could leave!”* (Danielle, IH)

#### Internal Coercion

For other patients, loss of control was mainly due to their critical mental condition at the time of admission, which deprived them of self-mastery and the ability to manage their lives. They therefore perceived hospital admission as their only possible choice to cope with the situation and regain control over their lives:

*“In those moments* [of psychotic decompensation]*, I’m somehow, yeah, I realise that I’m not myself anymore, but I keep trying to face up to them. I try to keep up the appearances, but then I just can’t do it anymore! So, in that situation, I’m just unable to say, ‘You have to hospitalise me. I can’t take it anymore!’ ”* (Nicole, IH)

*“I didn’t have much choice about being sectioned or about the treatment because, seeing that it wasn’t working, I was scared that the high-phase, low-phase cycle would never end! So, no. I didn’t really have a choice! (…) When I’m really in a manic phase, I can’t control myself! (…) I’m unmanageable!”* (Lisa, VH)

Some patients reported feelings of relief and hope on hospital admission, perceiving their hospitalisation as an opportunity to take a break, disconnect and refocus on themselves:

*“So, at that moment, I said to myself, ‘Okay. Since they’ve offered me this help’, I told myself, ‘Maybe they’ll take it seriously, and maybe somebody will find out what’s really going on’, because nobody had ever put words to it before!”* (Veronique, VH)

*“I was thinking that, maybe, well, like this, I’ll be able to get some rest. I’ll be able to think about me!”* (Noemi, VH)

Patients’ feeling of losing control due to their illness even led a minority of them to describe intervention by an external authority as necessary. Other stakeholders, such as professional caregivers and family members, were thus legitimate to exercise power over them and make decisions on their behalf. The restrictions eventually imposed on them by those stakeholders, throughout the hospitalisation process, were then perceived as fair and justified by the need to protect them from the negative consequences of their condition:

*“He* [the out-of-hours doctor on call] *did the right thing, actually, by sectioning me. In the end, it means that it needed to be done!”* (Luca, IH)

*“*[Regarding the restrictions on outings imposed on her by caregivers during her hospitalisation] *I think that, well, they were thinking about what was best for me… (…) they said, ‘Well, it’s for your own protection!’”* (Noemi, VH)

However, not all the patients who perceived high levels of *internal coercion* evoked the same patterns of experiences and feelings throughout their hospitalisation process. Indeed, after admission, some of them perceived strong negative external pressures, moving closer to the *external coercion* side of the experience and developing a feeling of *dual coercion*.

#### Dual Coercion

For some patients, the perceptions of external and internal pressures varied during the hospitalisation process and, at some points, overlapped. As mentioned above, these patients acknowledged that their mental condition was the primary reason for their loss of control over their lives and that hospitalisation was their only chance to restore that control *(internal coercion)*. However, once in hospital, they felt overwhelmed by the restrictions and the power exerted over them by an external authority *(external coercion)*, further increasing, rather than reducing, their feeling of disempowerment:

*“I’d never been there against my will before, but they sectioned me because I refused to take some of the treatments, because I didn’t want to be drugged like I was the first times, and they medically sectioned me under the pretext that I wasn’t capable of deciding that I didn’t want a medical treatment or something!”* (Nicole, IH)

Initial feelings of relief and hope, therefore, were quickly replaced by feelings of helplessness and fear, which intensified patients’ perceptions that they were no longer in control and should resign themselves to conforming to what the external authority expected of them:

*“So, when they talked to me about the section order, I was scared, I can tell you. I told myself that I’d never get out of there; that I wasn’t going to make it; that they’d stick me under guardianship again or something! I saw myself losing everything, my children, everything! So, I went along with them; I took my medications. And when they saw that I was going along with them, and that I was being a good little girl, well, they cancelled my section order a few days later!”* (Nicole, HV)

Hospital admission was therefore regarded as a form of protection but also a violation of patients’ autonomy and freedom—a contradiction that patients struggled to resolve, even months later:

*“Yes and no! Yes, because on one side, I was in a little box, and I was being kept there, and I was being monitored* [in a protective sense]. *It was mainly that! But, no, as well, because taking away my freedom was horrible! Because then I felt that I was nothing at all! So, when it comes to your self-confidence, it was awful!”* (Lisa, VH)

Most patients, however, felt hopeless and resigned to the idea of having a relapse and, thus, having to submit to this external authority once again. Only one of our 12 interviewees categorically rejected any possibility of another future hospitalisation, even if that entailed a risk of being formally and forcefully coerced:

*“From up there to* [psychiatric hospital X]*, no way. I’d be too scared! I think they’d have to take me there by force!”* (Jean, VH)

### Factors Affecting the Experience

Patients reported several factors that affected their perception of the balance between *internal coercion* and *external coercion* throughout the hospitalisation process and, thus, their final view of admission as a form of protection or violation. We categorised these factors into three themes: *the institutional context*, *relationships with professionals* and *the meaningfulness of the experience*. Although, for the sake of clarity, these themes are discussed separately, they were strongly interconnected throughout patients’ narratives. Moreover, the theme of *the meaningfulness of the experience*, while affecting patients’ experience of loss of control, was also a result of that loss, creating a vicious circle of reciprocal relationships.

#### The Institutional Context – *“I didn't belong there!”*

Psychiatric institutions’ organisational set-ups, environments, rules and practices affected patients’ experience of hospital admission and their feeling of being subject to the power of an external authority. Several patients reported feeling out of place on ward, considering it an inappropriate environment for them and their needs:

*“That wasn’t the right place for me!”* (Marie, VH)

*“Me, I’d have wanted to change ward. I’d have wanted to be on a ward where the patients weren’t so sick, in inverted commas, but they apparently thought that was where I should be, and there you go! But I didn’t feel like I was in the right place at all, on that ward!”* (Nicole, IH)

Difficulties began upon arrival *(admission)*. Hospital admission was often described as too rushed and lacking guidance. Some patients were experiencing their first admission and, therefore, were completely new to the hospital environment. Others had been hospitalised before but in different hospitals or on different wards. A lack of knowledge about the institutional context and its organisational set-up increased patients’ feelings of uncertainty and anxiety at a time when they were already facing an acute crisis:

*“It was pretty quick* [reception at the hospital] *because it was already the night shift, and so it was basically, bah, ‘We’ll explain all the rest tomorrow!” (…) When you are already anxious, it’s not really very reassuring when your only information is, ‘Well, go to bed, and we’ll tell you about the rest tomorrow!’ (…) And then there weren’t the little brochures that you’re supposed to receive on admission!”* (Alice, VH)

Daily life on the ward was depicted as challenging and by no means beneficial (*ward life).* Patients perceived their hospital stay as a disruption of their lives, depriving them of their daily routines and liberties:

*“What I remember as being most shocking was really (…) when I realised that I needed, for example—I had no stuff with me. I just wanted some toothpaste, and I couldn’t get any. But, there you go. It was a load of little things that accumulated, and you realise, in the end, that it’s a whole load of small things!”* (François, IH)

Several organisational and environmental elements were mentioned as making daily life on the wards particularly difficult. Interviewees often complained about the excessively high number of patients on wards and the close proximity of patients with very different conditions, often perceived as more severely ill and even dangerous:

*“I think that, well, I saw some things that I never expected to see in my life. Some of the other patients…There were times at night, honestly, when I was scared because I heard some of them arriving with the police. That was frightening!”* (Lisa, VH)

Hospital environments were often described as unwelcoming, unpleasant and noisy, and the time spent there was empty and boring. The lack of activities was often cited as problematic. Most patients wanted to feel active during their stay. Activities were recognised as important not only for killing time but, above all, for helping patients feel alive, regain self-confidence and socialise with others:

*“It was cold; it wasn’t very nice! (…) Then, what I find negative and what’s hard, (…) is that there are very, very few activities. You’re cut off from everything; you’re in a room; you don’t see anybody. I spent my time smoking cigarettes!”* (Laura, IH)

*“You’re worse off than in prison! You go round and round your room! I think that even in prison, they’ve got activities!”* (Marie, VH)

On the other hand, being in a comfortable and calm environment, surrounded by people with whom they could interact and exchange with positively, feeling active during the day but also having the possibility to withdraw and have some privacy when necessary, were all identified as improving the hospital experience and ward life:

*“I took care of myself. I went out in the morning and in the afternoon, I listened to music (…) we did painting too, there were workshops and all that! Yes, it’s a calm place! I had a room all to myself, too. So, sometimes there was a film to watch, sometimes I couldn’t, but there you go. But there was also respect for what I wanted to do! (…) Speaking to people, even patients, yeah! Just thinking about something else, that’s good too! (…) Even when it came to cleanliness, there was nothing to complain about; it was always clean!”* (Noemi, VH)

External authority was also perceived as more or less disempowering depending on the degree of flexibility and fairness with which hospital rules and practices were applied (*hospital rules and practices*). Inflexible rules led patients to feel *“… as if you were in prison!”* (Maude, VH), increasing their sense of de-subjectivation and loss of autonomy. Hospital rules and practices were thus perceived as primarily driven by the need to preserve order and control on the ward rather than the need to take care of the patients:

*“You get the impression that you are in a cage where you are only allowed to do certain things! A meal. Right, you’re allowed that. You’re allowed to eat in your room, or you have to eat out with everybody else, or you have to eat in the cafeteria. They’re all obligations, and you’re a bit of a puppet: you go where they want you to go!”* (Lisa, VH)

Several rules and practices were severely criticised for being unfair and humiliating, while others were unclear or were applied subjectively by professionals, generating feelings of uncertainty and tensions with staff:

*“I don’t know if it’s a standard procedure of theirs, but I found it a bit humiliating that they took all my clothes from me. So, they put a naked person in a room!” (*Danielle, IH)

*“There’s a very big difference depending on which nurses or doctors you ask, in fact! Somethings are allowed by some, but not allowed by others!”* (Laura, IH)

Hospital rules and practices were perceived as less coercive and more acceptable when professionals applied them flexibly and patients were able to understand their meaning as well as their provisional status. In these cases, rules could also be experienced as a useful part of the treatment itself, enabling patients to improve their condition:

*“So, the fact that* [the rules] *are explicit and that you don’t have to guess them by running into barriers! And that what’s coming next is predictable too. Like, it’s this way for the moment, but we’ll reassess things tomorrow or after tomorrow, and, yeah, in the end, you know that things can evolve and when! Then, well, yeah, being able to understand the reasons too! (…) It’s better than simply, ‘This is the way it is!’”* (Alice, VH)

*“I had lots of eating disorders. (…) So, the fact that I had to sit down at a table and eat—and there was also somebody who was watching me a bit too—and seeing who else had eaten and everything, well, that really did me good!”* (Veronique, VH)

#### Relationships with Professionals – *“In there, you're neither heard nor listened to!”*

The quality of the relationships with hospital staff was a key element of patients’ experience. Patients always reported that positive encounters with professionals were the most helpful element in coping with their mental health crisis and hospital restrictions. On the contrary, negative relationships marked the patients deeply, amplifying their perception of being submitted to a malevolent external authority. Five main elements were essential to defining a positive relationship with mental health professionals: *empathy*, *information*, *voice*, *respect* and *trust*. First, patients needed to feel that staff supported them, genuinely cared about them and listened to them empathetically (*being empathetically supported*). However, professionals were often described as unavailable due to the institutional context that overloaded them with too many patients and administrative tasks, but also to their lack of commitment towards the patients. Professionals often appeared to lack empathy and sensitivity, increasing patients’ sense of abandonment and helplessness:

*“I really believe it, and without being nasty, the nurses didn’t give a flying damn about us! We were there all day going round and around in circles. Nobody looked after us—absolutely nobody!”* (Nicole, IH)

*“Nobody looked after me for three weeks! So, yeah, completely abandoned! (…) I got more empathy—I got a thousand times more empathy—in prison than up there!”* (Jean, VH)

Interactions with staff were described as mainly being devoted to administering treatments and monitoring rather than to emotional support. Disrupting the established order of things was the only way to get their attention. Being quieter and less disruptive than other patients, on the contrary, further increased the risk of not being seen or taken into consideration.

*“You go there in the morning. Knock-knock. You take your treatment. They watch carefully to see that you’ve taken it. You go there at lunchtime—same thing. You go there in the evening—same thing. Overall, that’s more or less all the interactions you get from the three nurses who are there!”* (Marie, VH)

*“Nurses who are drowning in work, who can’t look after me because there are more extreme cases, because I’m not well, I don’t want to live anymore, et cetera, but I’m not giving anyone a hard time! (…) You just had to not make too much noise and you’d be forgotten about!”* (Jean, VH)

In their interactions with professionals, patients also wanted to be properly informed about their clinical situation, their treatment plan and the interventions they would undergo (*being informed*). Information increased patients’ sense of security, reducing the anxiety of uncertainty. Understanding the reasons for the restrictions imposed on them helped patients to accept and cope with them more easily. On the contrary, the lack of information and explanations increased their feelings of powerlessness and objectification:

*“So, I lived more serenely, more calmly, I’d say, because I’d understood* [the reason for the section order]*, and now I had to take the time; I had to settle down more!” (*Luca, IH)

*“Having information on what’s… how it’s going to happen, how* [the medication] *is going to be handled, you know? How I’m going to manage all that! (…) we’re talking about me, here! So, I’d really like to be aware* [of what’s going on]. *Yes, I’d have preferred that!”* (Lisa, VH)

Lack of information was the primary barrier to patient understanding and participation in decision-making. Indeed, besides being informed, patients also wanted to be recognised as valuable informants and to be actively involved in decision-making processes throughout their hospital stay (*having a voice and being actively involved*). Positive relationships with professionals were possible, even under coercion, if patients’ voices were considered seriously and the value of their lived experiences was acknowledged, thus reducing the power imbalance inherent in patient–caregiver relationships. The use of shared decision-making tools, such as Joint Crisis Plans, was recognised as being very useful for this:

*“…because there’s actually a nurse who takes the time to understand the situation, to discuss it with us—with me—and to write it all out over two or three interviews!”* (Alice, VH)

On the contrary, the delegitimisation of patients’ voices led not only to feelings of disempowerment but also to feelings of worthlessness and stigmatisation. Patients felt that, because of their mental condition, their points of view were less valuable and not worth being considered:

*“I told myself, ‘Well, they’re not listening to me anymore because they think I’m not all there, that there aren’t any short moments when I’m myself again, when I’m conscious!’ I wasn’t listened to. No, not at all!”* (Nicole, IH)

*“The patient’s word has a lot less value than, well, for example, for the treatment specifically (…) the* [somatic hospital’s] *letter carried more weight than my personal list of treatments, even though it* [somatic hospital’s letter] *was drawn from that* [personal list of treatments]*! But the letter was written out by a doctor and so it carried more weight than what I was telling them, even though my personal list was the source of it!”* (Alice, VH)

Feeling worthless and stigmatised were also the consequences of the lack of fairness and respect that patients perceived in their interactions with professionals* (being treated fairly and respectfully)*. Patients needed to feel that professionals saw the human being behind the disease. Disrespect and humiliation increased their perception of de-subjectivation and strongly affected patients’ self-esteem, self-worth and sense of dignity, making the entire hospital experience distressing and, in some cases, even traumatic:

*“I felt completely powerless there, and stripped of everything: of human respect, of privacy! (…) I felt as if I was nothing; I felt as if I was in the grasp of the doctors, of people who weren’t listening to me, who had no regard for me! (…) I’m considered crazy, in inverted commas! I know that—I don’t like the word, but that’s the way it is! I think it’s a label that had been put on me! And they don’t look after us during the day, we’re worthless, we go round in circles, we’re not listened to! So, that’s it. That’s a stay in* [psychiatric hospital Y], *eh!”* (Nicole, IH)

*“Even if you can affirm that a person has a high suicide risk, I don’t think that gives the right to—in the presence of several people—undress someone and leave them without any clothes* [in an intensive care room]*! (…) I don’t think that I’ll ever be able to forget what I lived through!”* (Danielle, IH)

Empathy, support, information and involvement, combined with fairness and respect, together fostered the development of trusting relationships (*trusting professionals)*. In order to be able to trust professionals, patients needed to perceive their closeness and feel recognised as individuals. All these interpersonal skills were identified as extremely helpful in terms of care:

*“But there are some others* [mental health professionals] *who I think I’ll remember all my life because they helped me so very much! (…) With those people, well, there was a listening ear and support, in fact! And they were really talking to me, not to the patient, but really to me, and that was super important, and I found it enriching! (…) That’s when I got closer to them, and that’s what helped me the most, in fact. And those are the people I sought out when things weren’t going well!”* (Lisa, VH)

However, study interviewees often perceived a lack of transparency among staff, and they consequently considered them unreliable and untruthful. When professionals were distrusted, patients were more prone to pretend and hide who they were or how they really felt for fear that this might backfire on them, further hindering the development of the therapeutic alliance. Distrust and pretending were thus described as the only way to cope with the authority and its control:

*“There are some sorts of things that you mustn’t say and, as a nurse, I know what they are, and so I was careful not to say them! And we know that its auto and hetero-aggression and…it’s especially that, in fact! (…) Yes, you have to be careful!”* (François, IH)

*“I wasn’t sleeping properly, but I never said so… (…) I never said anything about it because I didn’t know how people would understand what I was saying—how they would interpret what I was saying! I was scared to say anything! I’m afraid of being judged; I didn’t know whether they write a report!”* (Maude, VH)

#### The Meaningfulness of the Experience – *“I'm not sure that I was really better when I was discharged!”*

When patients perceived that their hospitalisation, or at least some aspects of it, was indeed appropriate to their needs and was truly helping to improve their condition, they were able to make sense of their experience and their feeling of coercion were lessened, thus increasing their satisfaction:

*“It wasn’t easy. I’m a little ashamed, but I think it’s what I needed at that moment! I think it was good. It also enabled me to calm down that state of crisis that I was in. So, no, I think it was necessary!”* (Veronique, VH)

As mentioned above, even though several patients perceived hospitalisation as the only way to resolve their mental crisis and regain control over their life, once in hospital, only a few of them actually found what they were looking for *(getting what was needed)*. Indeed, most of the interviewees felt that they had not got what they needed or expected out of their hospitalisation, and they thus struggled to find meaning in their experience, which left them with a strong sense of helplessness:

*“I had some contact with the doctors, and it was like, ‘Hello, how are you doing today? Are you taking your medications? Are you sleeping?’ I wasn’t expecting that. I was expecting that they’d say to me, ‘Missus X, can you tell us why you go off the rails like that? What sets off these crises?’ But I never had any of that and I asked myself what was the point of my—I don’t know how many—days in* [psychiatric hospital Y]*! Because I wanted to be healed!”* (Nicole, IH)

Patients found some hospital interventions particularly helpful and effective, such as filling out a Joint Crisis Plan or setting up a healthcare network and a post-discharge treatment plan *(receiving effective treatment)*:

*“They found a nurse who always, well, she comes to my place every week (…). A nurse also for the kids! (…) The children, they’re both monitored by a psychiatrist too. They also proposed couples therapy… (…) Yep, so I got out of hospital but, well, with good follow-up, it was very rich!”* (Noemi, VH)

*“And the fact that we’ve written out a Joint Crisis Plan, and thus knowing that I’ve got it, if ever, is reassuring too!”* (Alice, VH)

Otherwise, hospital treatment was mostly described as ineffective or even harmful. Some patients reported no positive changes in their mental health situation following hospitalisation, whereas for others, it even worsened their difficulties and was thus an experience they wanted to avoid in the future:

*“For me, in any case, it was a very bad experience, yeah: painful and bad and—I’ll say it again—apart from the meals, I get absolutely nothing out of it!”* (Jean, VH)

*“… there was something traumatising about it, anyway! So, the main consequence was that, well, once again, at the start of the year, the possibility of another hospitalisation came up, and on learning that the only place in the canton available was in* [psychiatric hospital Z]*, I refused to go there!”* (Alice, VH)

## Discussion

The main aims of this qualitative study were to explore voluntary and involuntary patients’ experience of coercion during their psychiatric hospitalisation and identify which factors, from their perspectives, most affected it. Our results showed that all the patients interviewed perceived the hospitalisation as an experience of loss of control over their lives due to either external or internal pressures, as well as to a combination of the two. These findings were in line with Beauchamp and Childress’ [41] definition of personal autonomy as being free from the external constraints and personal limitations that prevent making choices. When study participants felt overpowered by an external authority, hospitalisation was experienced as a violation of their personal autonomy. Conversely, if they perceived that it was their mental condition that was overwhelming them, hospital admission was described as the only possible means of regaining their autonomy. Several studies have highlighted this dichotomy [18, 42–49]. A previous qualitative study on the experience of involuntary hospitalisation used the category of ‘losing control’—similar to our first overarching theme of ‘loss of control’—to describe the results of the internal and external pressures faced by patients [50]. However, the existing literature was unclear on whether and how these two apparently opposite perceptions could co-exist and interact within the same person [51]. Indeed, to date, very few studies have reported on patients with ambivalent feelings about their experience of coercion [43, 49], and none have explained how those feelings were determined or how they unfolded during hospitalisation.

Our results showed that some people could experience both *internal* and *external coercion*—perceiving the same hospital stay as a simultaneous form of protection and violation—and that a variety of factors could affect the balance between these opposite feelings throughout the hospital stay. These factors can modulate the perception of coercion, either by decreasing it and enabling the patient to regain control over their life, or by increasing it and inducing a feeling of *dual coercion*. Addressing these factors through targeted interventions could therefore alter this balance increasing patients’ sense of empowerment and fostering their recovery process.

Patients reported a number of contextual and relational factors that determined the ‘coercive context’ [3] within which their experience of loss of control was shaped. Many described hospital as an inhospitable environment, with rigidly dictated rules and daily routines that were too far removed from their usual lives. The lack of information about how their new environment would function and was organised often created anxiety and insecurity, as did cohabitation with other patients with very different conditions. This revealed that even psychiatric inpatients were not immune from experiencing stigmatisation towards other people with mental disorders. Several previous studies have stressed the potentially disempowering effect of the institutional context for both voluntary and involuntary patients. Johansson and Lundman (2002) described involuntary patients’ feelings of being overwhelmed by the inflexibility of hospital rules [18]. Indeed, professionals frequently use rules and routines as a means of informally coercing patients to adhere to treatments and social norms [52]. McGuinness et al. (2013) reported on the importance of admission procedures, especially in terms of the information provided, as determinants of the hospital experience [53]. Potthoff et al. (2022) emphasised how institutional settings and spatial surroundings influenced the way voluntary patients felt about the perceived psychological pressures weighing on them [54].

Patients’ experience is directly influenced by the institutional context but also indirectly influenced by how that context affects mental health professionals’ style, attitudes and availability. The inflexibility of hospital rules, routines and schedules may lead staff to adopt a more patronising and disciplinary style in their interactions with patients rather than a supportive one. Ward organisation, with frequent case overload, staff shortages and heavy administrative burdens can affect the amount and quality of the time professionals can devote to their patients. Finally, how the ward surroundings are designed and decorated can enhance patients’ feelings of being under surveillance, increase their physical and interpersonal separation from professionals and make it more difficult to find quite spaces in which to talk to them in private. Contextual factors are thus preconditions for developing good relationships [55] and should be considered more seriously.

Relationships with professionals were the key determinants of patients’ experience of coercion in our study. The five main elements identified as essential for building positive, non-coercive staff–patient relationships were empathy, information, voice, respect and trust. When these elements were lacking, professionals were perceived as mere controlling authorities, sometimes even malevolent and abusive ones, whose main task and interest was maintaining order on the ward. This result was in line with previous research about the essential role that interpersonal processes play in coercion [2, 54, 56, 57]. Several authors have emphasised the importance of giving patients information and involving them in decision-making processes, whether they are being admitted voluntarily or involuntarily [58–60]. Nevertheless, a lack of information and cooperation has been reported very often in similar qualitative studies, showing how much still needs to be done to effectively implement shared decision-making in psychiatry [19]. Cooperation, empathy, respect and trust form the base on which a therapeutic alliance can be built [61, 62]. Positive interactions with staff have the potential to make coercion and loss of autonomy more acceptable to patients [56, 63], whereas non-helpful relationships increase their feeling of being coerced [55]. Perceiving relationships with professionals as being humiliating, disrespectful and dehumanising increases patients’ fear and distrust towards them, leading patients to avoid mental health services in the future [64]. Negative relationships with professionals also strongly affect patients’ view of self, diminishing their self-esteem and confining them in an illness-dominant identity [65–68]. Several patients interviewed for the present study reported feelings of de-subjectivation, helplessness, worthlessness, distrust and stigmatisation resulting from their interactions with staff. A strong sense of hopelessness was also particularly evident among the patients in the *dual coercion* group, many of whom appeared resigned both to future relapses and their subsequent unavoidable submission to external authority.

Feelings of disempowerment and helplessness were also reported by our interviewees as the consequence of their struggle to make sense of the whole hospital experience. This was often perceived as useless, ineffective and, in some extreme cases, a hindrance to their recovery. Being able to make sense of such a difficult experience helps patients to reconcile themselves with it and integrate it into their life story and recovery process, thus decreasing the associated feeling of coercion. On the contrary, when the experience is perceived as extraneous and meaningless, the associated feeling of coercion grow and foster a ‘sealing over’ recovery strategy characterised by avoidance and denial [53, 69]. Patients adopting this ‘sealing over’ recovery style, instead of an ‘integrative’ one, are at an increased risk of future involuntary readmission [70]. Contextual and relational factors may affect how meaningful the hospital experience was and, consequently, patient satisfaction with it. Indeed, for a hospital experience to be perceived as meaningful and satisfying, patients need to feel that their daily life on the ward is purposeful, not empty, that the treatment provided to them is tailored to their needs, not rigidly standardised, and that their relationships with professionals and other patients are helpful and supportive, not abusive and disrespectful. Making sense of their hospital experience is, therefore, both a determinant and a result of their experience of coercion, generating a vicious circle difficult to disentangle. Moreover, these results confirmed the negative association between perceived coercion—as well as perceived fairness, information, respect, empathy and shared decision-making—and treatment satisfaction, as reported by the scientific literature [28, 71].

The present study found no apparent differences according to admission status. The experience of coercion and the factors influencing it did not substantially differ between voluntary and involuntary patients, confirming that how coercion was contextually and relationally implemented was more important than the coercive measures themselves [56, 72]. To quote Danielle, one of our study interviewees, *“I think there is always a reason to be section! But it’s always in the manner* [of doing that]! *Manu militari! We can give ourselves every right to do that militarily, or to do it more humanely!”.*

Finally, all participants expressed great satisfaction with the study’s interview process. Many of them reported feeling relieved at having finally had the chance to talk about their experience in detail and in a neutral space. The presence of a service-user researcher during the interviews was also greatly appreciated. Participants reported feeling reassured and more at ease, thanks to this presence. Furthermore, sharing their experience with someone who had gone through something similar made them feel heard and fully understood.

## Implications for Clinical Practice

Based on the present study’s results and the existing literature on this topic, we offer some recommendations for reducing patients’ experience of coercion in clinical practice. At admission and throughout the first few days of hospitalisation, special care must be taken to provide patients with comprehensive and comprehensible information. This should help patients to understand clearly where they are, why they are there, how the ward is organised and why, what its rules and timetables are, what they should expect over the following days and, if possible, approximately how long their hospital stay will last. If formal coercion is applied, patients must be informed as soon as possible about the reasons justifying the measure and its duration. Furthermore, both voluntary and involuntary patients must be clearly informed about their rights, including their right to appeal in the event of coercive measures. Hospitals should be organised into small units, within which ward rules should be kept to a minimum, and daily routines should be as flexible and individualised as possible. A more ‘family-like’ and less institutionally managed form of daily life should be fostered, and a variety of activities should be offered to promote patients’ self-confidence and recovery, reducing their feelings of disruption and loss of ‘normality’ [20]. A high staff-patient ratio should be ensured, and administrative tasks should be minimised in favour of staff continuity and availability.

Particular attention should be paid to the training of mental health professionals and the development of their interpersonal and communication skills to ensure respectful and empowering patient care. Professionals should empathetically support patients, take the time to talk and listen to them, acknowledge patients’ expertise and work with them collaboratively to foster partnerships and ensure that patients’ specific needs and expectations are considered. Patients should be provided with the relevant information and resources they need to better deal with their condition, increasing their options and fostering hope. Mental health professionals must be respectful, reliable, helpful and genuinely concerned if they wish to promote patient trust and therapeutic alliance. Finally, professionals should help patients regain a positive sense of self, move beyond their role of ‘patients’ and make sense of their whole hospital experience, thus reducing its potentially negative effects. The scientific literature has also identified these ingredients as essential components of recovery-oriented interventions [73]. Such interventions should therefore be implemented throughout the hospital stay, to decrease patients’ feeling of loss of control and reduce its negative effects. Patients should also be provided with post-discharge support programmes and recovery plans to help them cope with the consequences of their hospitalisation, regain control over their lives and promote the recovery process.

## Study Strengths and Limitations

The present study’s main strength was its qualitative design, which provided an in-depth understanding of how and why patients felt coerced during the psychiatric hospitalisation process. Its second strength was the involvement of a service-user researcher at each stage of the project’s implementation. We believe that the service-user researcher’s presence during the interviews greatly fostered participants’ openness and reduced the power imbalance between them and the interviewers. It also made the other researcher—unmistakably identified as a mental health professional—appear less threatening.

Some limitations should be noted, however. Due to the limited number of participants interviewed, our study results may not be fully generalisable, and a selection bias cannot be excluded. Indeed, patients who refused to participate in the study could have reported different experiences. However, because we sought high variability in patients’ experience of coercion during the interview recruitment phase and thanks to the richness of the narratives collected, we believe that this limitation did not have a major impact on our results. Finally, since our phenomenological description arise from the meeting between researchers and participants, it has to be considered as one interpretation and we cannot exclude the possibility of other potential complementary descriptions [33].

## Conclusions

The present study provided an in-depth understanding of patients’ perspectives of their experience of coercion during voluntary and involuntary psychiatric hospitalisation. Our results showed that patients mainly perceived their admission to hospital as an experience of loss of control due to both internal and external pressures. The institutional setting within which the inpatient stay takes place, the attitudes of ward staff towards their patients and the possibility for patients to find a meaning to the whole experience are all factors that can mitigate or reinforce this feeling of loss of control. Increasing policy-makers’ and clinicians’ awareness about these factors is of paramount importance for developing the skills and strategies needed to address them, reinforcing patient empowerment, reducing patients’ feeling of coercion and improving their well-being.

## Data Availability

Data will be made available upon reasonable request.
